# Audiological and psychological assessment of tinnitus patients with normal hearing

**DOI:** 10.3389/fneur.2022.1102294

**Published:** 2023-01-12

**Authors:** Yelin Park, Seung-Ho Shin, Sung Wan Byun, Zoo Young Lee, Ho Yun Lee

**Affiliations:** Department of Otorhinolaryngology-Head and Neck Surgery, Ewha Womans University School of Medicine, Seoul, Republic of Korea

**Keywords:** tinnitus, normal hearing, tinnitus distress, sound intolerance, psychiatric symptom

## Abstract

**Introduction:**

This study was performed to assess identifiable abnormalities in tinnitus patients with normal hearing.

**Methods:**

The medical records of subjective non-pulsatile tinnitus patients with normal hearing confirmed by conventional pure-tone audiometry who visited our tinnitus clinic between March 2020 and May 2022 were reviewed. The loudness discomfort level (LDL), extended high-frequency hearing loss (EHFHL), summating potential (SP)/action potential (AP) ratio, distortion product otoacoustic emission (DPOAE), thresholds of auditory brainstem response (ABR) wave V, somatic modulation, and psychiatric symptoms, such as anxiety, depression, and stress were evaluated by questionnaires.

**Results:**

Decreased LDL (*n* = 48, 59.8%) was the most frequent finding, followed by EHFHL (*n* = 29, 35.4%), increased SP/AP ratio (*n* = 27, 32.9%), psychiatric symptoms (*n* = 24, 29.3%), decreased DPOAE (*n* = 17, 20.7%), somatic modulation (*n* = 8, 9.8%), and increased ABR threshold (*n* = 3, 3.7%); 75.6% of patients had one or more of these findings. The presence of psychiatric symptoms was independently associated with the Tinnitus Handicap Inventory (THI) score.

**Conclusion:**

Tinnitus in patients with normal hearing may be accompanied by a combination of various subclinical abnormal audiological findings. However, the presence of psychiatric symptoms alone was independently associated with tinnitus distress.

## 1. Introduction

Tinnitus is the conscious awareness of a tonal or composite noise without an identifiable cause (1). Changes in the central auditory pathway caused by peripheral auditory deafferentation due to age-related hearing loss or noise-induced hearing loss may explain the tinnitus percept in most situations because tinnitus usually occurs following hearing deterioration. The risk factors are similar for age-related hearing loss and tinnitus, with both aging and cardiovascular disease as prognostic factors (2).

However, some tinnitus patients show normal hearing in conventional audiometry and do not feel any subjective hearing loss or aggravation of hearing loss along with new-onset tinnitus. Despite conventional audiometry findings within the normal range, about 10–15% of subjects have self-report hearing loss (3). A cross-sectional study based on the National Health and Nutrition Examination Survey 1999–2002 reported that confusion/memory, self-reported hearing difficulty, pain/tingling in hands/feet, balance problems, and diabetes were common in patients with persistent tinnitus and normal audiometric threshold (3). With regard to risk factors associated with tinnitus in subjects with normal hearing, the Korea National Health and Nutrition Examination Survey (KNHANES) showed that tinnitus is associated with female sex, ischemic heart disease, dyslipidemia, noise exposure, and depression (4).

Tinnitus disorders are present in tinnitus patients with tinnitus and accompanying emotional, attentional, or cognitive problems (1). Tinnitus distress results from integrating various brain networks involving the limbic, auditory, hypothalamus, etc., (5). A recent review highlighted the role of the triple network in tinnitus distress (6). Tinnitus distress is also aggravated by accompanying hyperacusis (7). Taken together, these findings suggest that various changes in the auditory, emotional, and somatosensory systems may be involved in the generation and maintenance of tinnitus. These changes include decreased loudness discomfort level (LDL), extended high-frequency hearing loss (EHFHL), increased summating potential (SP)/action potential (AP) ratio, decreased distortion product otoacoustic emission (DPOAE), altered wave V thresholds of auditory brainstem response (ABR), somatic modulation, and accompanying psychiatric symptoms (4, 7–28). In addition, various subclinical auditory dysfunctions may be hidden. These have been reported in studies in subjects with normal hearing and tinnitus. On the other hand, as we gained more clinical experience in identifying auditory abnormalities in tinnitus patients with normal hearing, we observed that there always exists not just one auditory abnormality at one time in many patients with tinnitus with normal hearing. In many cases, several abnormal findings occurred simultaneously. However, to our knowledge, it remains unclear which characteristics are most common in these patients and whether they exist alone or simultaneously. In addition, insufficient information is available regarding which factors are most closely associated with tinnitus distress.

It is important to know which etiologies are more common in cases of normal hearing and tinnitus because, when these patients visit a tinnitus clinic, more successful treatment outcomes may be achieved by preparing for more common causes in advance and first addressing these causes. Therefore, this study was performed to assess the identifiable audiological abnormalities and psychiatric problems in tinnitus patients with normal hearing.

## 2. Materials and methods

### 2.1. Patients and documented variables

The medical records of tinnitus patients who visited a tinnitus clinic at a tertiary university hospital between March 2020 and May 2022 were screened and reviewed. The inclusion criteria were all pure-tone threshold not exceeding 25 dB from 250 to 8 kHz and non-pulsatile subjective tinnitus. Exclusion criteria were as follows: brain malignancies; and neurological deficits. Based on review of medical records, we documented the patients' age, sex, accompanying symptoms (aural fullness, headache, dizziness, attention problems, temporomandibular/neck pain, sleep disturbance, and history of exposure to loud noise), accompanying diabetes mellitus, and/or hypertension.

The Tinnitus Handicap Inventory (THI) score was determined for all patients. THI score ≥38 was considered to indicate the presence of moderate distress. Psychiatric symptoms were assessed using several questionnaires, including Beck's Depression Inventory (BDI), Beck's Anxiety Inventory (BAI), and the Brief Encounter Psychosocial Instrument (BEPSI). Anxiety was considered present in cases with BAI score ≥22. Depression was defined as BDI score ≥16. BEPSI score ≥1.8 was taken to indicate the presence of stress.

### 2.2. Psychoacoustic tests and physical examinations

For audiological evaluation, pure-tone audiometry, speech audiometry, ABR, electrocochleography, and tinnitogram consisting of pitch (Hz), loudness (dB SL), and minimal masking levels (MMLs) were performed. The mean pure-tone hearing threshold was calculated as the arithmetic means of hearing at 0.5, 1, 2, and 4 Hz. For bilateral tinnitus, the hearing threshold of the right side was used to calculate the pure-tone threshold. DPOAE was measured using the Neuro-Audio system (Neurosoft, Ivanovo, Russia), and the presence of DPOAE was defined as a signal-to-noise-ratio > 6 dB at five of eight f2 frequencies up to 8 kHz. Click ABRs were recorded with Navigator Pro software (Biological Systems Co., Mundelein, IL, USA). The threshold of wave V and wave V amplitude at the 90-dB click stimulus were documented.

A threshold exceeding 30 dBnHL was defined as an increased threshold of wave V of ABR (29). Decreased LDL was defined as LDL ≤ 90 dB at two or more frequencies (7). Increased SP/AP ratio was defined as a ratio ≥0.4 (30). EHFHL was defined as hearing threshold >15 dB in patients in their 20 s, 50 dB in those in their 30 s, 55 dB in those in their 40 s, 65 dB in those in their 50 s, and 75 dB in those in their 60 s (31).

Somatic modulation test was performed as described previously (32). Patients were considered to have positive somatic modulation if their tinnitus was modulated by at least one of the neck or jaw maneuvers.

### 2.3. Statistical analysis

Descriptive analysis was conducted to evaluate patient characteristics. Numerical data were compared between groups using Student's *t*-test. Pearson's correlation analysis was performed to analyze correlations between pairs of numerical variables. Binary logistic regression analysis with the ENTER method was performed to determine which abnormal findings significantly affected THI and tinnitus loudness. All analyses were performed using SPSS for Windows ver. 27.0 (IBM Corp., Armonk, NY, USA). In all analyses, *p* < 0.05 was taken to indicate statistical significance.

## 3. Results

### 3.1. Patient characteristics

**Figure 1:**
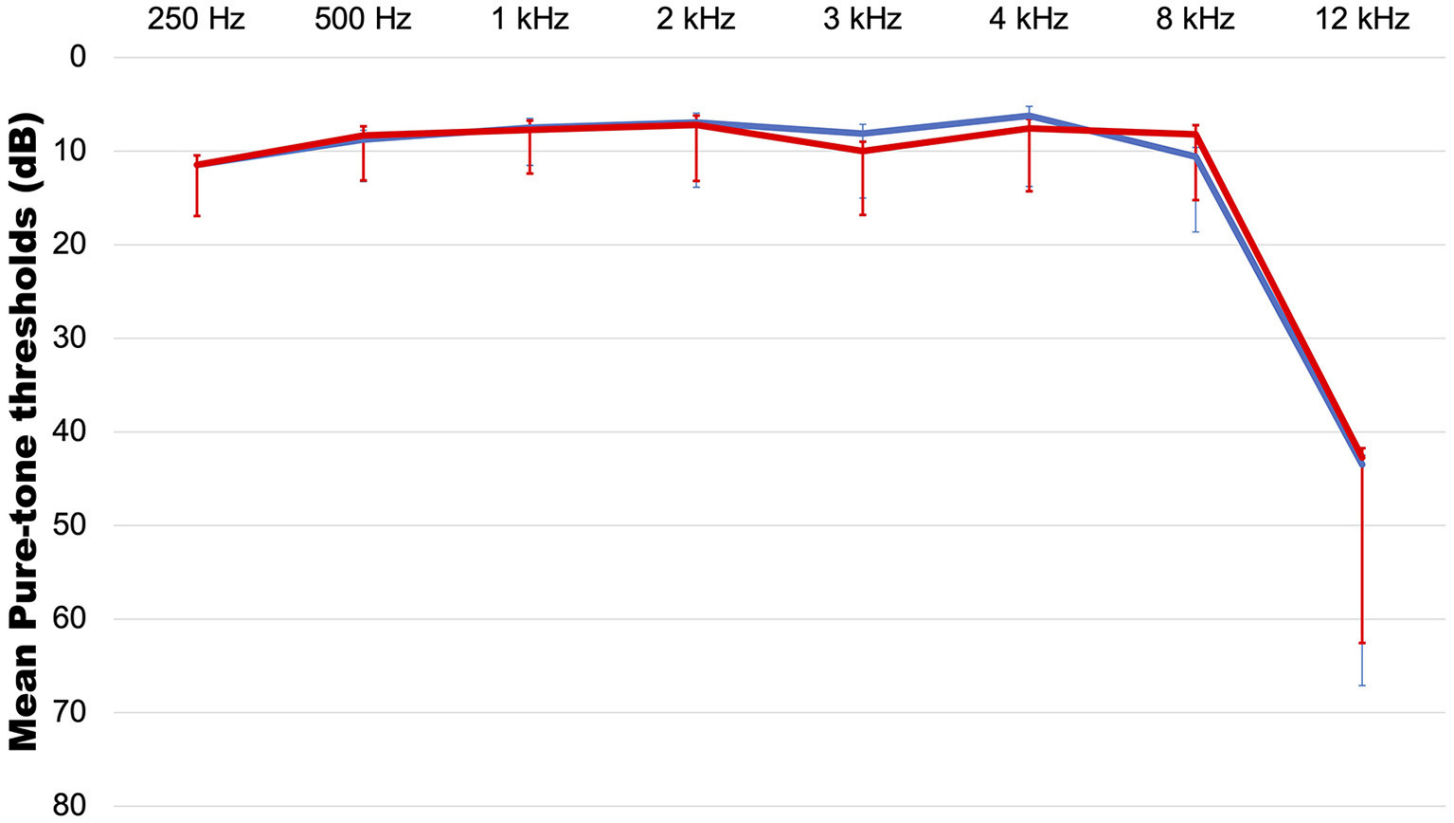
The average audiogram. red line: right side, blue line: left side. Error bar indicates standard deviation.

Eighty-two patients consisting of 28 males (34.1%) and 54 females (65.9%) with a mean age of 37 years (range: 14–64 years) were included in the study. The symptom duration was 13.87 months (range: 0.5–240 months). The mean hearing levels were 7.78 ± 3.94 dB on the right (range: 2–17 dB) (Figure 1) and 7.71 ± 4.08 dB on the left (range: 2–18 dB). With regard to laterality, 47.5% (*n* = 39) had unilateral tinnitus, 43.9% (*n* = 36) had bilateral tinnitus, and the remaining patients (*n* = 7, 8.5%) complained of non-lateralized tinnitus. The most common accompanying symptom was aural fullness (*n* = 35, 42.7%), followed by headaches and hyperacusis (*n* = 25, 30.5%), sleep disturbance (*n* = 22, 26.8%), dizziness (*n* = 20, 24.4%), neck pain (*n* = 16, 19.5%), and attention difficulty (*n* = 9, 11.0%). Hypertension (*n* = 8, 9.8%), diabetes (*n* = 1, 1.2%), and thyroid disease (*n* = 5, 6.1%) were reported as comorbidities, and 12.2% (*n* = 11) of patients had a history of exacerbation after noise exposure. Based on the results of the questionnaires, 65.2% (*n* = 15/23) of the patients had a moderate or higher level of stress, while 26.1% (*n* = 6) and 22.0% (*n* = 18) had anxiety and depression, respectively.

### 3.2. Abnormal findings in tinnitus patients with normal hearing

Decreased LDL (*n* = 49, 59.8%) was the most common possible etiology, followed by EHFHL (*n* = 29, 35.4%), increased SP/AP ratio (*n* = 27, 32.9%), psychiatric symptoms (*n* = 24, 29.3%), decreased DPOAE response (*n* = 17, 20.7%), somatic modulation (*n* = 8, 9.8%), and increased ABR threshold (*n* = 3, 3.7%) (Table 1).

**Table 1:** Abnormal audiological findings observed in tinnitus patients with normal hearing.

**Number of abnormal findings**	**Abnormal DPOAE**	**Decreased LDL**	**Psychiatric symptoms**	**Somatic modulation**	**Increased SP/AP ratio**	**Extended high frequency hearing loss**	**Increased ABR thresholds**	**Total**
0	0	0	0	0	0	0	0	15 (18.3%)
1	2 (10.5/11.8%)	8 (42.1/16.3%)	2 (10.5/8.3%)	0	3 (15.8/11.1%)	4 (21.1/13.8%)	0	19 (23.2%)
2	4 (22.2/23.5%)	14 (77.8/28.6%0	6 (33.3/25.0%)	1 (5.6/12.5%)	5 (27.8/18.5%)	3 (16.7/10.3%)	3 (16.7/100%)	18 (22.0%)
3	5 (25.0/29.4%)	18 (90.0/36.7%)	9 (45.0/37.5%)	3 (15.0/37.5%)	11 (55.0/40.7%)	14 (70.0/48.3%)	0	20 (24.4%)
4	4 (50.0/23.5%)	7 (87.5/14.3%)	5 (62.5/20.8%)	4 (50.0/500%)	6 (75.0/22.2%)	6 (75.0/20.7%)	0	8 (9.8%)
5	2 (100.0/11.8%)	2 (100/4.1%)	2 (100/8.3%)	0	2 (100.0/7.4%)	2 (100.0/6.9%)	0	2 (2.4%)
Total	17 (20.7%)	49 (59.8%)	24 (29.3%)	8 (9.8%)	27 (32.9%)	29 (35.4%)	3 (3.7%)	100%

Among the items evaluated, 24.4% (*n* = 20) of patients were positive for three abnormal findings, followed by 23.2% (*n* = 19) with one abnormal finding, 22.0% (*n* = 18) with two, 9.8% (*n* = 8) with four, and 2.4% (*n* = 2) had five abnormal findings (Table 1). There were no abnormal findings in 18.3% (*n* = 15) of cases.

The number of abnormal findings showed a weak positive correlation with tinnitus awareness (*r* = 0.341, *p* = 0.002) and effect of tinnitus on life (*r* = 0.231, *p* = 0.037). However, the THI showed no correlation with the number of abnormal findings (*p* > 0.05).

### 3.3. Influence of abnormal findings on THI and tinnitus loudness

Regression analysis showed that only the presence of accompanying psychiatric symptoms was significantly associated with THI≥38 (Table 2). None of the etiological factors examined showed a significant association with tinnitus loudness (data not shown).

**Table 2:** Results of binary logistic regression analysis of the factors affecting Tinnitus Handicap Inventory in tinnitus patients with normal hearing.

**Variables**	**B**	**S.E**.	**Sig**.	**EXP (B)**	**95% CI**	
					**Lower**	**Upper**
Decreased DPOAE response	0.136	0.628	0.829	1.145	0.334	3.924
Decreased loudness discomfort level	0.051	0.553	0.927	1.052	0.356	3.108
Accompanying psychiatric symptoms	−1.706	0.589	0.004	0.182	0.057	0.576
Presence of somatic modulation	−1.048	0.865	0.226	0.351	0.064	1.910
Increased SP/AP ratio	0.009	0.540	0.986	1.009	0.351	2.907
Increased ABR wave V threshold	−0.788	1.429	0.581	0.455	0.028	7.486
Extended-high frequency hearing loss	0.467	0.555	0.400	1.596	0.538	4.736
Constant	2.452	1.696	0.148	11.614		

## 4. Discussion

Decreased LDL showed the highest incidence in this study (*n* = 48, 59.8%), followed by various audiological findings suggestive of subclinical auditory dysfunction in patients with normal hearing and tinnitus. In most cases, patients had multiple abnormal findings, while 18.3% of patients did not show any prominent abnormal findings. In addition, the presence of psychiatric symptoms was independently associated with THI. None of the audiological findings examined in this study showed a significant relation to tinnitus distress.

Patients with concomitant tinnitus and hyperacusis complained of more severe tinnitus distress, and a criterion for the co-occurrence of LDL ≤ 90 dB at two or more frequencies can be applied to predict accompanying hyperacusis (7). As described above, decreased LDL was the most common abnormal finding in this study. Similar to this study, in our previous study with 194 tinnitus patients, 26.3% had subjective symptoms, and 68.4% also showed lower LDL by the same criteria (7). Therefore, we assumed that hyperacusis is not a unique symptom observed only in tinnitus patients with normal hearing but seems to be a common symptom in tinnitus patients regardless of the hearing level.

On the other hand, decreased sound tolerance was persistent in adolescents with normal hearing and tinnitus who did not recover during 1-year follow-up (8). To diagnose decreased sound tolerance, thorough history taking, audiological assessment, and psychological evaluation are necessary to exclude the possibility of misophonia (9). In a recent study, where tinnitus patients and a control group of patients with normal or symmetric hearing loss were enrolled, patients with unilateral tinnitus had significantly lower LDLs than the control group (10). However, those with bilateral tinnitus showed no difference in LDLs compared to the control group. The authors interpreted that the decreased sound tolerance may reflect hidden cochlear damage, but it leads to unilateral tinnitus only and bilateral involvement depends on the hearing status (10). These authors discussed a previous study reporting differences in quantitative electroencephalography (qEEG) findings between unilateral tinnitus and bilateral tinnitus, with the former showing increased gamma-band activity in the contralateral parahippocampal and auditory cortex, and the latter showing an association with delta activity in the ventrolateral prefrontal cortex (11).

EHFHL is quite common, occurring in 64% of subjects aged 18–65 years, and can begin even in the early 20 s for males (12). Risk factors include noise exposure, drugs, infection, premature aging, heredity, and head trauma. EHFHL is associated with a high risk of future hearing loss and may also affect speech recognition (13). In addition, EHFHL is correlated with cognitive performance, regardless of tinnitus (14). EHFHL can also cause tinnitus in subjects with normal hearing and may appear normal in conventional audiometry, especially in young patients under 35 years old, and tinnitus patients were shown to have a worse extended high-frequency (EHF) threshold than controls (15). Worsening of EHFHL was observed during 1-year follow-up in patients with decreased sound tolerance and persistent tinnitus (8).

Auditory nerve fibers with high thresholds and low spontaneous firing rates are preferentially destructed in cochlear synaptopathy after aging or noise exposure (33). Reduced amplitude of ABR wave I and increased ABR V/I amplitude ratio are the most common predictors of cochlear synaptopathy in animals (16). However, ABR wave I is not often measured in humans, and the amplitude of wave V varies widely (16, 34)—these may limit the applicability of ABR to detect cochlear synaptopathy in humans. Various techniques have been tried to overcome the shortcomings of conventional ABR and increase the detection rate of synaptopathy in humans. As a result, a small latency shift of wave V in masked ABR was found to be a better indicator of cochlear synaptopathy in humans and mice (35). In addition, the quantification of envelope following responses (EFR) evoked by the application of rectangular amplitude modulation tone predicted word recognition better than conventional sinusoidal amplitude modulation (36). The other study by Vasilkov et al. also reported the optimal stimulation paradigms for this measurement method (37). In addition to ABR, electrocochleography (ECoG) is also available to detect hidden hearing loss. The results of ECoG are usually interpreted as audiological evidence of endolymphatic hydrops, and are also regarded as cochlear synaptopathy because of the similarity to ABR in that the summating potential comes from hair cells and the AP is equivalent to wave I of ABR (17).

A previous study showed a higher SP/AP ratio in patients with normal hearing and tinnitus than in those without tinnitus (18). Cochlear hydrops or cochlear synaptopathy may have been mixed etiologies in our patients.

Subclinical auditory dysfunction can also be assessed by DPOAE or ABR. Some reports provided evidence of increased latency of ABR wave I in subjects with normal hearing with tinnitus compared to those without tinnitus (19). Contrary to ABR wave I, which often shows an increased latency and decreased amplitude in tinnitus patients with normal hearing, alterations in waves III and V are inconsistent (38). Some reported the increased latencies of waves III and V (39). However, our previous study observed a shortening of latency in waves III and V in patients with bilateral tinnitus compared to the normal control (40). For abnormal DPOAE, reduced DPOAE amplitude is common in tinnitus patients with normal hearing, suggesting subclinical cochlear degeneration. These observations suggest that outer hair cells (OHCs) play an important role in tinnitus generation. However, others suggested that, although OHC dysfunction is associated with tinnitus, changes in OHCs do not always lead to the generation of tinnitus (20). That is, OHC dysfunction is not the only determinant of tinnitus (21). DPOAE and transient evoked OAE (TEOAE) did not differ according to the presence or absence of tinnitus in subjects with normal hearing (18). Another study similarly showed that DPOAE amplitudes did not differ according to the presence of tinnitus and/or hyperacusis, and were instead affected by EHF hearing thresholds (22). Reduced DPOAE is common in subjects with normal hearing regardless of accompanying tinnitus.

Some tinnitus patients may modulate their tinnitus by head and neck maneuvers or eye movement, regardless of hearing loss (23). Somatosensory tinnitus is a condition associated with head and neck pain or problems, such as temporomandibular joint disorders and bruxism (24). The following conditions indicate the presence of somatosensory influence: neck or jaw pain that appears simultaneously with tinnitus; neck/jaw symptoms that are simultaneously aggravated with tinnitus; head or neck trauma preceding tinnitus; varying pitch, loudness, and/or location; and discrepancies in audiogram and unilateral tinnitus (25). Disinhibition or unfamiliar somatosensory input to the dorsal cochlear nucleus may be regarded by the brain as changed auditory perception even in subjects with normal hearing (26). The percentage of patients with somatic modulation in this study was merely 9.8%, which was much lower than our previous study results (61.7%). We assumed that this substantial difference was due to differences in clinical settings or the experiences of audiologists (32).

Of the psychiatric symptoms, higher rates of depression were seen in tinnitus patients with normal hearing even after adjusting for other confounders, such as age, sex, past medical history, and noise exposure (4). These patients have a higher prevalence of depression and anxiety than those without tinnitus, and the severity of psychiatric symptoms is correlated with tinnitus distress (27). Although younger tinnitus patients tend to have normal hearing compared to older patients, the rates of self-reported depression and stress showed no differences according to age (28). In our study, about a quarter of subjects with normal hearing showed psychiatric symptoms, which was less than initially expected. However, this was the sole, independent prognostic factor for tinnitus distress, suggesting that control of psychiatric symptoms is the most important consideration for relieving tinnitus distress.

The auditory dysfunction may be addressed by correction of decreased sound tolerance. To our knowledge, there have been no randomized controlled trials of medications for treatment of hyperacusis. Therefore, gabapentin, anticonvulsants, anxiolytics, or antidepressants may be chosen empirically based on the clinician's clinical experience. We usually recommend that patients with hyperacusis alone avoid sound and use ear protection because of their heightened sensitivity to sound. For those with both tinnitus and decreased sound tolerance, listening to broadband noise, such as pink noise at a well-tolerated level that does not induce discomfort, may be appropriate (41). In addition, sound therapy is recommended to induce habituation to tinnitus. Total masking or partial masking based on tinnitus retraining therapy can be chosen based at the physician's discretion. Next, patients should be checked for recurrent vertigo or fluctuating hearing loss to exclude the possibility of endolymphatic hydrops. In addition, noise exposure history and ABR results should also be confirmed to avoid missing the possibility of cochlear synaptopathy. Due to the possibility of somatic tinnitus, various treatments, including physical therapy and muscle relaxants, may also be considered. It should be emphasized that tinnitus does not have a single cause, so treatment must be multifaceted. Of the various treatments available, the psychiatric symptoms should be treated first.

This study had several limitations. The relatively low number of 82 patients was too small to analyze the overall etiologies of tinnitus in patients with normal hearing. We checked EHF hearing loss at 12 kHz, but a more diverse analysis would have been possible if we had tested up to 16 kHz. However, it was impossible to acquire additional data because the audiometer in our hospital did not support frequencies in this range. In addition, no abnormal findings were observed among the items reviewed in 18.3% of the patients. These patients may have had subclinical abnormalities that could not be detected by questionnaires or audiological tests (42).

## 5. Conclusion

Various subclinical auditory abnormalities were observed in tinnitus patients with normal hearing, and most cases showed several abnormalities simultaneously. However, only the presence of psychiatric symptoms was independently associated with THI.

## Data availability statement

The raw data supporting the conclusions of this article will be made available by the authors, without undue reservation.

## Ethics statement

The studies involving human participants were reviewed and approved by Ewha Mokdong University Hospital. Written informed consent for participation was not required for this study in accordance with the national legislation and the institutional requirements.

## Author contributions

HL: conceptualization, data analysis and methodology, writing-original draft, and writing-review and editing. YP: writing-original draft, data analysis, and writing-review and editing. S-HS and SB: writing-review and editing. ZL: data curation, data analysis, and methodology. All authors contributed to the article and approved the submitted version.
